# Trauma-Informed Pedagogy and Refugee Student Engagement in Australian Schools: A Systematic Review

**DOI:** 10.1007/s40653-026-00903-5

**Published:** 2026-05-08

**Authors:** John Kole

**Affiliations:** Deakin University, Burwood, Australia

**Keywords:** Trauma-informed pedagogy, Refugee education, Culturally responsive teaching, Student engagement, School inclusion, Educational wellbeing

## Abstract

Refugee-background students in Australian schools often experience trauma that significantly affects their wellbeing, engagement, and academic success. While trauma-informed education has gained prominence internationally, limited research has explored how such approaches are conceptualised and implemented in Australian contexts, particularly within structured frameworks such as Multi-Tiered Systems of Support (MTSS) and School-Wide Positive Behaviour Support (SWPBS). This systematic review investigates how trauma-informed pedagogy supports refugee students’ engagement and wellbeing in Australian schools and examines the extent to which multi-tiered frameworks are integrated into these practices. Comprehensive searches were conducted across seven academic databases between September and October 2025, following PRISMA 2020 guidelines. Ten peer-reviewed empirical studies published between 2015 and 2025 met the inclusion criteria. Data were extracted and thematically synthesised using an inductive approach to identify recurring concepts and patterns. Five interrelated themes emerged from the synthesis: the centrality of relationships and pedagogical care; cultural responsiveness and identity affirmation; teacher capacity and professional learning; systemic and structural barriers to implementation; and the emerging alignment of trauma-informed practice with MTSS and SWPBS frameworks. The findings reveal that while relational and culturally responsive approaches are widely recognized as essential for supporting refugee students, there remains limited empirical evaluation of whole-school or system-level implementation. Overall, the review concludes that trauma-informed education in Australia is characterised by innovation but lacks consolidation. Stronger policy alignment, sustained teacher professional learning, and longitudinal research are needed to embed trauma-informed pedagogy within multi-tiered, culturally responsive, and relational frameworks that promote resilience, equity, and belonging for refugee-background learners.

## Introduction

Australia has experienced a steady increase in refugee arrivals, many of whom are children and adolescents who have endured significant trauma before, during, and after migration (Department of Home Affairs (DHA), 2023; Miller et al., 2022). For example, children held in immigration detention have exhibited high rates of mental health disorders, with posttraumatic stress disorder (PTSD) exceeding 30%, anxiety at 44%, and depression at 32% (Blackmore et al., 2020; Tomasi et al., 2022). These outcomes are shaped by cumulative trauma, resettlement challenges, and social isolation across all stages of displacement (Guo et al., 2023), profoundly impacting refugee students’ mental health, academic achievement, and school engagement (Lembke et al., 2024; Miller et al., 2021; Odhiambo, 2025).

Refugee students’ experiences of schooling in Australia are shaped by intersecting histories of displacement, adaptation, and institutional practices. Many enter schools with interrupted or limited prior education, unfamiliarity with Australian pedagogical norms, and varying levels of English proficiency, factors that can contribute to academic difficulty and disengagement (Miller et al., 2018; Molla, 2025). However, research consistently shows that these challenges are compounded by racialisation, stereotyping, and deficit-based assumptions within school contexts, which undermine students’ sense of belonging and recognition as capable learners (Baak, 2019; Baak et al., 2025).

Studies highlight how experiences of othering, discrimination, and cultural misrecognition erode emotional safety and trust, which are essential conditions for learning after trauma. Baak (2019) demonstrates how South Sudanese heritage students frequently encounter racialized positioning that exposes the limits of inclusion in practice, while Tippett et al. (2024) show how schools struggle to reconcile competing discourses of equity and equality. These tensions shape the nature and consistency of support provided to refugee students and may inadvertently reproduce disadvantage. Informal learning opportunities, supportive peer relationships, and caring pedagogical practices, conceptualised as pedagogical love, can foster emotional safety, a sense of belonging, and sustained engagement, thereby contributing to more positive educational trajectories.

To understand how these experiences connect with learning, it is important to consider school engagement. According to Molla (2024), school engagement refers to the degree of involvement, attention, interest, and enthusiasm that students and, in some cases, parents demonstrate toward education and learning. It encompasses emotional, cognitive, and behavioural investment in schooling and is reflected in how students feel, think, and act in relation to their academic work. School engagement includes both academic and social dimensions, influencing not only students’ achievement but also the quality of their relationships within the learning environment. As a multidimensional construct, it is shaped by individual characteristics, social contexts, and broader policy factors that determine the depth and quality of students’ participation in education (Appleton et al., 2008; Fredricks et al., 2004).

Schools play a pivotal role in supporting refugee students; however, educators often lack trauma-informed frameworks to meet their complex needs (Barrett & Berger, 2021; Lembke et al., 2024). Childhood trauma is consistently linked to impaired academic, behavioural, and socio-emotional functioning, including attention difficulties, aggression, hyperactivity, anxiety, and low self-esteem (Perfect et al., 2016; Weems et al., 2009). Students with PTSD are particularly vulnerable to learning and behavioural difficulties. As a result, scholars argue that trauma-informed practices are essential for creating safe, predictable, and relational learning environments (Chafouleas et al., 2016; Nadeem & Ringle, 2016).

Multi-tiered frameworks such as School-Wide Positive Behaviour Support (SWPBS), also referred to internationally as Positive Behavioral Interventions and Supports (PBIS), and Multi-Tiered Systems of Support (MTSS) provide structured approaches for addressing diverse student needs through universal, targeted, and intensive levels of support (Sugai & Horner, 2006; Weist et al., 2018). PBIS is defined by the Centre on PBIS as an evidence-based, three-tiered framework designed to support students’ behavioural, academic, social, emotional, and mental health needs, while creating positive, predictable, equitable, and safe learning environments (Centre on PBIS, 2026). MTSS, in turn, offers a flexible framework for embedding trauma-informed practices, with Tier 1 focusing on predictability, cultural responsiveness, and relational safety; Tier 2 supporting social and emotional learning and self-regulation; and Tier 3 providing individualised, and often therapeutic, interventions (Brown, 2025).

However, the alignment between SWPBS or MTSS and trauma-informed education is neither automatic nor ideologically neutral. Kim and Venet (2025) caution that PBIS implementations frequently prioritise behavioural compliance and standardisation, which can undermine relational and contextual approaches to trauma. Venet (2023) further contends that trauma-informed education must be explicitly grounded in equity, with a focus on power dynamics, racialisation, and structural inequities that shape how student behaviour is interpreted and addressed in schools. Absent this critical lens, tiered behaviour systems risk reinforcing deficit-based narratives and contributing to the disproportionate disciplining of marginalised students, particularly those from refugee backgrounds (Liasidou, 2024; McIntosh & Goodman, 2016). Expanding on these critiques, Thomas (2025) argues that genuine integration between SWPBS and trauma-informed education demands a commitment to empathy, relationship-building, and equity across all levels of implementation.

Together, these critiques suggest that although SWPBS and MTSS are preventive, safety-oriented frameworks that appropriately include behaviour regulation to protect students and staff, their trauma-informed implementation requires careful integration of relational, culturally responsive, and equity-oriented practices. Without such integration, behavioural expectations may inadvertently marginalise refugee-background learners or misinterpret trauma-related behavioural expressions (Kim & Venet, 2025; Liasidou, 2024; McIntosh & Goodman, 2016; Sugai & Horner, 2006; Watson & Astor, 2025). In this sense, SWPBS/PBIS and related multi-tiered frameworks are most effective when they are used not as stand-alone behaviour management systems, but as structural platforms through which trauma-informed pedagogy can be enacted across whole-school settings.

Trauma-informed pedagogy refers to educational approaches that recognise the prevalence and effects of trauma on learning and prioritise safety, trust, relational connection, and empowerment within classroom practice (Chafouleas et al., 2016; Nadeem & Ringle, 2016). In refugee education contexts, trauma-informed pedagogy extends beyond behaviour management to include culturally responsive, relational, and strengths-based practices that acknowledge students’ lived experiences of displacement and resettlement (Venet, 2023). Such approaches emphasise predictable learning environments, emotionally supportive teacher–student relationships, and inclusive pedagogical strategies that promote belonging, wellbeing, and engagement. Establishing this conceptual foundation is critical to understanding how trauma-informed educational practices can support refugee students’ participation and success in Australian school settings.

The central research question guiding this systematic review is: *How is trauma-informed pedagogy conceptualised and implemented to support refugee students’ engagement and wellbeing in Australian schools?* To address this question, the review critically examines peer-reviewed research on trauma-informed educational practices within Australian school settings, with a particular focus on their integration into multi-tiered intervention frameworks. It evaluates the quality of existing evidence, identifies key gaps in implementation, and synthesises findings to provide evidence-informed recommendations for strengthening trauma-responsive approaches that enhance refugee students’ engagement, wellbeing, and academic success.

## Methodology

### Search Strategy

This review is an intervention-focused systematic review conducted in accordance with the PRISMA 2020 guidelines (Page et al., 2021). While PRISMA served as the primary reporting framework, the review also aligns conceptually with the Campbell Collaboration standards for social and educational intervention reviews, given its focus on school-based trauma-informed interventions.

Comprehensive searches were conducted by the author between September and October 2025 across PsycINFO, Ovid MEDLINE, ERIC, A+ Education, Web of Science Conference Proceedings Citation Index–Science, ProQuest Dissertations, and Google Scholar to capture both peer-reviewed and empirical grey literature. While grey literature was considered to ensure breadth and inclusivity, only empirical studies reporting primary data were included; non-empirical and opinion-based publications were excluded. Search terms included combinations of: (“trauma-informed” OR “trauma-responsive ” OR “trauma-aware” OR “trauma-informed pedagogy” OR “trauma-informed practice” ) AND (refugee OR “asylum seeker” OR “migrant student” OR “new arrival” OR “humanitarian entrant”) AND (“multi-tiered systems of support” OR MTSS OR “school-wide positive behaviour support” OR SWPBS OR “positive behavioural interventions and supports”) OR PBIS AND (engagement OR participation OR attendance OR inclusion OR wellbeing OR “social-emotional learning”) AND (Australia OR “Victorian school ” OR “Australian education” OR “Australian context”).

Studies published between 2015 and 2025 were included. This timeframe was selected because 2015 marks a pivotal shift in global and Australian refugee education policy following the escalation of the international displacement crisis (Phillips & Spinks, 2013; United Nations High Commissioner for Refugees (UNHCR ) 2016). It also corresponds with the emergence of more conceptually coherent and empirically grounded trauma-informed education frameworks (Chafouleas et al., 2016; Miller et al., 2018). Limiting the search to this decade ensured that the review captured the most contemporary developments in trauma-informed practice, including the growing integration of MTSS and SWPBS models (Kim & Venet, 2025; Maynard et al., 2019), as well as recent Australian research addressing refugee students’ mental health, wellbeing, and engagement (Miller et al., 2022; Ziaian et al., 2018). Overall, this period provides the most relevant window in which trauma-responsive, multi-tiered school interventions have been clearly defined, widely implemented, and rigorously examined.

Eligible studies were required to examine trauma-informed pedagogy or school-based interventions, focus on refugee students in Australian schools (either exclusively or within mixed cohorts), and report multi-level trauma-informed approaches involving teachers, parents, and/or students. Exclusion criteria were studies conducted outside Australia, research not involving refugee populations, non-empirical papers, and studies conducted in specialist therapeutic settings. Both qualitative, quantitative, and mixed-methods research across all school levels (preschool–secondary) was included to capture the breadth of available evidence.

### Screening and Selection

Following the completion of comprehensive database searches, all retrieved records were imported into EndNote for reference management and the systematic removal of duplicates. The de-duplicated dataset was then exported to Rayyan, an online systematic review management platform, to facilitate structured screening, coding, and record tracking in accordance with PRISMA 2020 guidelines (Page et al., 2021).

Title, abstract, and full-text screening were conducted systematically by the reviewer to remove irrelevant records based on predefined inclusion criteria, thereby ensuring methodological rigour and consistency. Data extraction and coding were subsequently completed using Microsoft Excel, employing a standardised extraction matrix to promote transparency, accuracy, and traceability throughout the review process.

The initial search yielded 500 records. After removing 26 duplicates, a total of 474 titles and abstracts were screened for relevance against the inclusion criteria (empirical focus, trauma-informed pedagogy, Australian school context, and relevance to refugee populations). Of these, 417 records were excluded for not meeting inclusion criteria. Fifty-seven full-text articles were retrieved and assessed for eligibility. Of these, forty-seven studies were excluded based on predefined criteria: non-Australian context (*n* = 18), non-empirical or conceptual design (*n* = 12), absence of trauma-informed pedagogical focus (*n* = 9), and lack of relevance to refugee student populations (*n* = 8). Ten studies met all inclusion criteria and were retained for final synthesis. These exclusion categories and counts are summarised in the PRISMA 2020 flow diagram to enhance transparency and reproducibility of the study selection process.

**Fig. 1:**
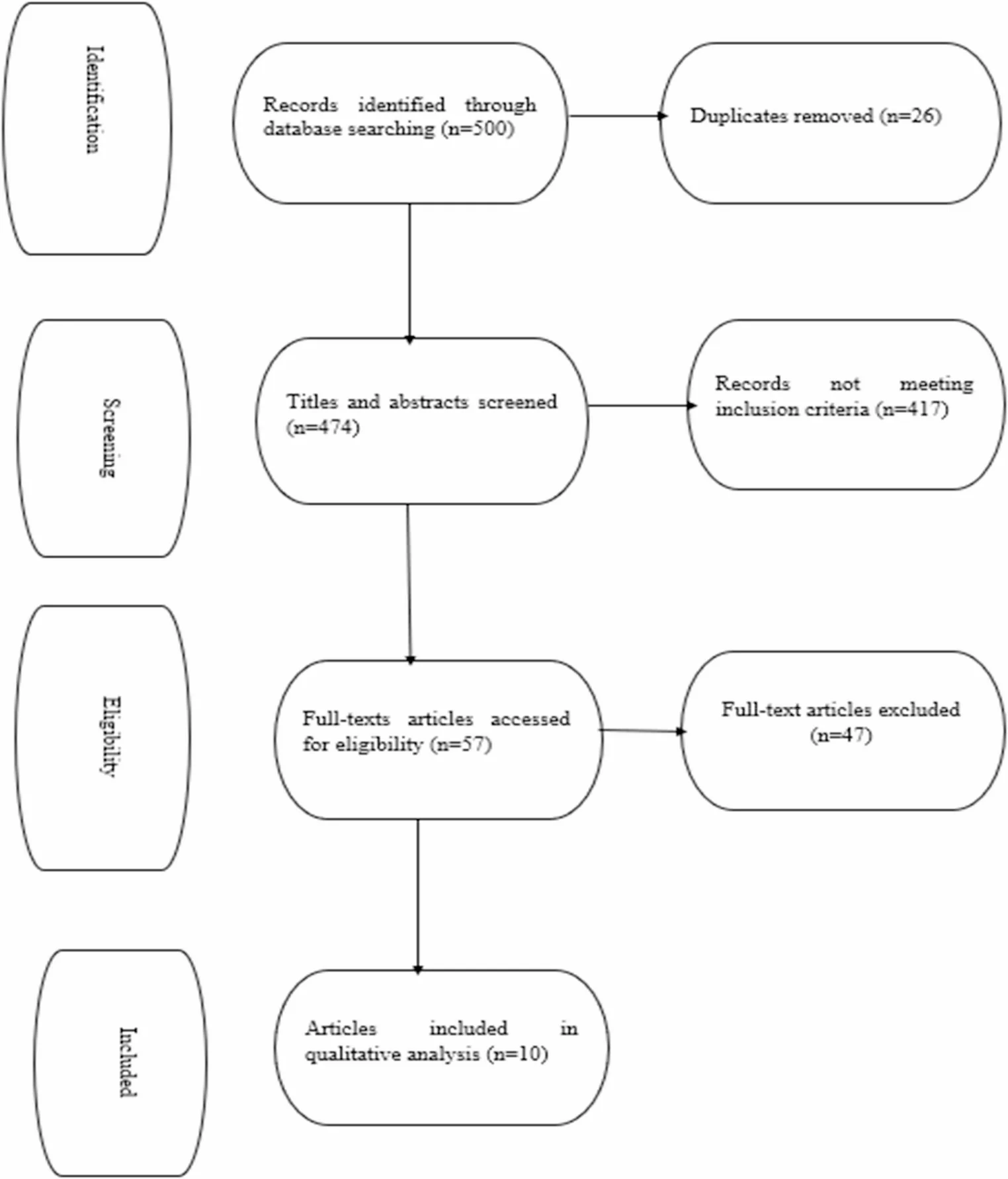
Flowchart of the screening process

The screening process was conducted in two stages: Title and abstract screening, in which studies clearly unrelated to the topic or outside the inclusion parameters were excluded. Full-text review, which involved detailed evaluation of methodology, context, and relevance to the research aims. Figure 1 provides a visual summary of the screening and selection workflow.

### Data Extraction and Coding

Data extraction and coding were conducted by the author in accordance with PRISMA 2020 methodological guidelines (Page et al., 2021). A standardised data extraction template was developed to ensure transparency, reliability, and replicability across studies. Each record was reviewed and coded according to the following categories: (a) author (s); (b) research methodology; (c) participant demographics; (d) type of trauma experienced; (e) study design; (f) intervention type; (g) key findings and outcomes; and (h) study limitations (see **Table ****1**).

The author utilised the Rayyan systematic review platform to facilitate and manage the data coding process. This tool enabled the systematic organisation, categorisation, and analysis of studies throughout the review. Predefined inclusion and exclusion criteria were applied consistently across all databases, and a subset of records was re-examined to ensure alignment with the established inclusion parameters. To enhance credibility, a methodological advisor with expertise in trauma-informed and refugee education reviewed a purposive subset of studies (*n* = 3) during the full-text screening and early data extraction phase to verify alignment with eligibility criteria and coding consistency.

A systematic and reflexive research approach was adopted, incorporating a structured critical appraisal of methodological quality using the Standard Quality Assessment Criteria for Evaluating Primary Research Papers (Kmet et al., 2004). Appraisal outcomes informed the interpretation of study quality, strengths, and limitations during synthesis rather than serving as a basis for study exclusion. The appraisal was used to contextualise the synthesis by identifying methodological strengths and limitations across the included studies, thereby informing the weight given to particular findings and areas of caution in interpretation.

The empirical literature within the Australian context did not provide sufficient evidence to warrant the inclusion of MTSS and SWPBS as distinct coding categories. A comprehensive search revealed no peer-reviewed empirical studies directly examining the intersection of refugee education and multi-tiered or schoolwide behavioural frameworks in Australian schools. Consequently, these frameworks were incorporated as conceptual lenses informing the overall design and interpretation of the review rather than as discrete analytical categories.

The coding framework was iteratively refined to remain grounded in the data rather than guided by preconceived assumptions, ensuring alignment with the principles of inductive thematic synthesis (Thomas & Harden, 2008). To enhance transparency and analytic rigour, the number of studies contributing to each theme was recorded during coding, enabling frequency mapping of thematic support across included studies. Coding categories emerged inductively from the empirical evidence, reflecting dominant themes related to trauma-informed practices, teacher perspectives, and inclusion strategies. Nonetheless, the discussion integrates MTSS and SWPBS as emerging frameworks with potential relevance for future implementation and research in trauma-informed education. This interpretation is supported by recent scholarship (Brown, 2025; Liasidou, 2024; Newton et al., 2023), which underscores the growing alignment between trauma-informed approaches and multi-tiered, schoolwide systems of support.

**Table 1:** Data extraction and coding summary

Author(s) & Year	Methodology	Participants	Intervention Type	Key Findings	Limitations
Lembke et al., 2024	Systematic review	Review of 28 studies (global and Australian)	Trauma-informed school models	Five components: safety, relationships, empowerment	Limited Australian evidence
Barrett & Berger, 2021	Qualitative interviews	*n* = 25 teachers	Trauma-informed professional learning	Improved empathy and engagement	Small sample
Wrench et al., 2018	Participatory ethnography	*n* = 40 students	Arts-based trauma-informed practice	Increased belonging and identity	Limited generalisability
Odhiambo, 2025	Mixed methods	*n* = 60 teachers; *n* = 15 students	Trauma-informed inclusive framework	Trust predicted engagement	Cross-sectional
Baker & Naidoo, 2024	Qualitative case study	*n* = 15 students	Trauma-informed mentoring	Improved belonging	Tertiary focus
Wiseman & O’Gorman, 2017	Qualitative case study	Single Australian school (exact numbers not specified)	Everyday trauma-informed practice	Need relational and culturally responsive pedagogies	Single site
Miller et al., 2018	Systematic review	35 Australian studies	School inclusion and trauma-informed supports	Gaps in trauma-informed teacher training	Limited longitudinal data
Miller et al., 2021	Qualitative study	~ 30 participants	Inclusion and wellbeing supports	Systemic inequities and deficit discourses	No intervention evaluation
Kaukko & Wilkinson, 2020	Ethnographic case study	*n* = 20 students	Informal trauma-informed supports	Relationships key for adaptation	Informal context; not trauma-informed programs
Kaukko et al., 2021	Comparative case study	*n* = 22 teachers & *n*=18students, in Finland and Australia	Pedagogical love framework	Care enables safety and engagement	Small sample

*MTSS* Multi-Tiered Systems of Support, *SWPBS* School-Wide Positive Behaviour Support, n = sample size

## Key Findings and Analysis

A synthesis of the reviewed studies revealed five interrelated themes that shape the implementation and impact of trauma-informed pedagogy for refugee-background students in Australian schools. These themes illustrate the intersection of relational, cultural, and systemic factors influencing students’ engagement, wellbeing, and sense of belonging. The reviewed body of research, comprising qualitative, mixed-method, and review-based studies published between 2015 and 2025, reflects an emerging yet uneven field of practice.

In addition to these thematic insights, the review identified prevailing methodological approaches, core theoretical orientations, and persistent research gaps that collectively advance understanding of trauma-informed education within the Australian refugee schooling context.

### Methodological Designs and Approaches

The reviewed studies predominantly employed qualitative and mixed methods designs, reflecting the exploratory, relational, and context sensitive nature of trauma-informed educational research (Barrett & Berger, 2021; Kaukko & Wilkinson, 2020; Odhiambo, 2025). Qualitative methodologies, such as ethnographic case studies, semi structured interviews, focus groups, and participatory observations, were especially prevalent, enabling in depth exploration of refugee students’ lived experiences and teacher practices in diverse school settings (Miller et al.,^2021^; Wiseman & O’Gorman, 2017; Wrench et al., 2018). These approaches foreground narrative depth and contextual meaning, privileging participants’ voices over statistical generalisability.

Several studies, including (Baker & Naidoo 2024; Odhiambo 2025), adopted interpretivist and participatory frameworks grounded in relational ethics, compassion, and care. Such approaches align with the ethics of care and pedagogical love, emphasising collaboration, reflexivity, and empowerment as both methodological and pedagogical commitments. By positioning participants as co-researchers, these studies extend trauma-informed inquiry beyond observation to include agency, recovery, and social justice dimensions.

In contrast, review-based and conceptual analyses (Lembke et al., 2024; Miller et al., 2018) synthesised empirical and theoretical literature to map trends, identify systemic gaps, and examine the translation of trauma-informed principles into multi-tiered or whole-school frameworks. Together, this methodological diversity—from deep qualitative inquiry to conceptual synthesis—reflects a maturing and methodologically plural field that recognises the need to integrate personal, relational, and systemic perspectives in refugee education research.

### Theoretical Orientations

The reviewed studies draw on four interrelated theoretical orientations that collectively inform trauma-informed pedagogy for refugee-background students in Australian schools: trauma theory; ecological systems theory; social constructivism and culturally responsive pedagogy; and the ethics of care and pedagogical love. Together, these frameworks emphasise that effective trauma-informed education must integrate psychological safety, systemic coherence, cultural validation, and relational care to support students’ recovery, engagement, and learning (see **Table ****2**).

Trauma theory underpins much of the literature, highlighting how adversity disrupts emotional regulation, cognitive functioning, and social relationships, and how safety, trust, and relational attunement are foundational to learning (Barrett & Berger, 2021; Lembke et al., 2024). Ecological systems theory (Bronfenbrenner, 1979) extends this lens by situating trauma within interconnected family, school, community, and policy contexts, underscoring the need for coordinated, multi-tiered supports that align classroom practices with broader school and system-level responses (Miller et al.,^2018^; Miller et al., 2021).

Social constructivist and culturally responsive perspectives conceptualise learning and recovery as relational and culturally situated processes, where inclusive curricula and meaningful relationships foster belonging and participation (Kaukko & Wilkinson, 2020; Wiseman & O’Gorman, 2017; Wrench et al., 2018). Complementing this, the ethics of care and pedagogical love foreground empathy, moral responsibility, and relational justice, positioning care and recognition as central to equitable education for refugee students (Baker & Naidoo, 2024; Kaukko et al., 2021; Odhiambo, 2025).

Together, these orientations show that trauma-informed pedagogy is most effective when psychological, ecological, cultural, and ethical dimensions intersect within coherent multi-tiered frameworks such as MTSS and SWPBS.

**Table 2:** Theoretical orientations of reviewed studies

Theoretical orientation	Aligned studies
Trauma theory	(Barrett & Berger 2021; Lembke et al., 2024; Odhiambo 2025)
Ecological systems theory	(Lembke et al., 2024; Miller et al., 2018; Miller et al. 2021)
Social constructivism and culturally responsive pedagogy	(Kaukko & Wilkinson 2020; Miller et al., 2021; Wiseman and O’Gorman 2017; Wrench et al., 2018)
Ethics of care and pedagogical love	(Baker &Naidoo 2024; Barrett & Berger 2021; Kaukko et al. 2021; Odhiambo 2025)

Individual studies may align with more than one theoretical orientation

### Key Themes

A synthesis of the reviewed studies identified five interrelated themes that illuminate how trauma-informed pedagogy is conceptualized and enacted for refugee-background students in Australian schools. To strengthen interpretive credibility, the thematic synthesis was informed by documentation of the number of studies contributing to each theme, supporting transparent and evidence-grounded discussion of patterns across the literature. These themes, centred on relational and pedagogical care, cultural responsiveness and identity affirmation, teacher capacity and professional learning, systemic and structural barriers, and emerging multi-tiered frameworks, illustrate the interplay of interpersonal, cultural, and institutional factors shaping educational engagement and wellbeing (see **Table ****3**). They highlight both the strengths and gaps within the evolving landscape of trauma-informed education in Australia.

**Table 3:** Frequency of studies contributing to each theme

Theme	Number of studies	Supporting studies
Relationships and pedagogical care	6	(Barrett & Berger 2021; Kaukko et al., 2021; Odhiambo 2025; Wiseman & O’Gorman 2017; Kaukko & Wilkinson 2020; Baker & Naidoo +^2024^)
Cultural responsiveness and identity affirmation	5	(Wrench et al., 2018; Miller et al., 2021; Kaukko & Wilkinson 2021; Wiseman & O’Gorman 2017; Odhiambo 2025)
Teacher capacity and professional learning	4	(Barrett & Berger 2021; Miller et al., 2018; Odhiambo 2025; Miller et al. 2021)
Systemic and structural barriers	4	(Miller et al. 2021; Odhiambo 2025; Lembke et al., 2024; Miller et al., 2018)
Emerging multi-tiered frameworks	2	(Lembke et al., 2024; Odhiambo 2025)

Frequencies represent the number of included studies (*n* = 10) contributing to each theme. Additional conceptual and international literature is discussed in the manuscript to contextualise the emerging multi-tiered frameworks theme but is not included in these counts

### Theme 1: The Centrality of Relationships and Pedagogical Care

Across the reviewed literature, strong teacher–student relationships and an ethics of care consistently emerge as the cornerstone of trauma-informed practice. Six studies contributed to this theme (*n* = 6), emphasising that trust, empathy, and “pedagogical love” are not peripheral but central to creating emotionally safe and inclusive learning environments (Kaukko & Wilkinson, 2020; Kaukko et al., 2021). These relational qualities help refugee-background students navigate feelings of alienation, loss, and displacement while rebuilding confidence and agency in educational spaces (Barrett & Berger, 2021; Odhiambo, 2025; Wiseman & O’Gorman, 2017; Wrench et al., 2018).

Such findings align with broader trauma theory, which positions relational attunement as a key mechanism for recovery and self-regulation. When teachers demonstrate consistency, care, and emotional responsiveness, students are better able to trust adults and engage meaningfully in learning. Thus, trauma-informed pedagogy extends beyond instructional strategies to encompass an ethical and relational commitment to human dignity and belonging.

### Theme 2: Cultural Responsiveness and Identity Affirmation

Cultural responsiveness is identified as a defining dimension of effective trauma-informed education. Five studies contributed to this theme (*n* = 5), demonstrating that refugee-background students experience belonging and continuity when their languages, histories, and cultural identities are valued within classroom environments. Evidence further indicates that inclusive practices, including multicultural curriculum design, community partnerships, and recognition of students’ prior experiences, can counteract the dislocation and marginalisation many refugee learners face (Barrett & Berger, 2021; Kaukko & Wilkinson, 2020; Kaukko et al., 2021; Odhiambo, 2025; Wrench et al., 2018).

However, the literature also reveals tensions between policy rhetoric and practice. While Australian educational policies advocate inclusion and equity, Eurocentric curricula and institutional norms often perpetuate cultural invisibility and deficit framings of refugee learners (Miller et al., 2021). These inconsistencies underscore the need for trauma-informed frameworks that explicitly integrate cultural validation and identity affirmation as central pillars of practice rather than supplementary goals.

### Theme 3: Teacher Capacity and Professional Learning

Teacher preparedness and ongoing professional learning represent both a key strength and a persistent challenge within trauma-informed education. Four studies contributed to this theme (*n* = 4), highlighting both progress and gaps in teacher capacity building. While many educators express commitment to trauma-informed values, evidence indicates that professional learning opportunities are frequently fragmented or limited in scope, resulting in uneven implementation across school contexts. Teachers commonly report uncertainty about applying trauma-informed principles in cross-cultural settings and balancing academic expectations with emotional and wellbeing support for refugee-background students (Barrett & Berger, 2021; Miller et al., 2018; Miller et al., 2021; Odhiambo, 2025).

This uneven access to sustained professional development contributes to inconsistent practice, often reliant on individual teacher initiative rather than coherent institutional frameworks. Strengthening teacher capacity through structured, ongoing, and contextually relevant professional learning is therefore essential for ensuring fidelity, sustainability, and scalability of trauma-informed approaches across diverse school environments.

### Theme 4: Systemic and Structural Barriers to Implementation

Despite increasing recognition of trauma informed education’s value, several studies reveal that its implementation is often fragmented, under resourced, and disconnected from broader systemic reform. Four studies contributed to this theme (*n* = 4), highlighting structural constraints that limit sustainable implementation. Research indicates that inclusion and wellbeing policies are frequently undermined by rigid curricular structures, limited funding, and racialized disciplinary practices that disproportionately affect students from refugee backgrounds (Miller et al., 2021; Odhiambo, 2025). Additional evidence identifies inconsistencies in school resourcing, gaps in teacher training, and limited policy coherence as barriers to embedding trauma-informed approaches at scale (Miller et al., 2018; Lembke et al., 2024).

These systemic constraints hinder the institutionalisation of trauma informed education and reduce its long term sustainability. Collectively, the literature underscores that effective implementation requires leadership commitment, policy alignment, and cross sector collaboration. A whole school or multi-tiered approach is therefore necessary to ensure trauma informed education functions as a systemic, equity oriented framework supporting wellbeing, inclusion, and learning.

### Theme 5: Emerging Alignment with Multi-Tiered Frameworks (MTSS and SWPBS)

Theme 5 identified an emerging but underdeveloped alignment between trauma-informed pedagogy and multi-tiered frameworks, with only two included studies contributing directly to this theme (*n* = 2) and additional conceptual and international literature used to contextualise these findings. Although no Australian studies have directly examined the integration of MTSS or SWPBS within refugee education, emerging scholarship highlights their potential to embed trauma-informed practices at scale.

Emerging Australian research suggests that multi-tiered frameworks provide a structured organisational architecture for coordinating universal, targeted, and intensive supports across school systems, thereby strengthening consistency and implementation coherence (e.g., Brown, 2025; Eastman et al., 2025; Newton et al., 2023; Norrish & Brunzell, 2023). However, empirical evidence remains limited, particularly in relation to trauma-informed and refugee-specific applications.

More broadly, MTSS and SWPBS are preventive, schoolwide frameworks designed to support safe and inclusive learning environments through coordinated academic, behavioural, and wellbeing supports. Direct empirical research examining their implementation within refugee education remains scarce. International scholarship indicates that these frameworks offer a promising organisational structure for coordinating supports for refugee learners, particularly those affected by trauma and resettlement challenges (e.g., Garbacz et al., 2018; Kim & Venet, 2025; Lembke et al., 2024; Liasidou, 2024; McIntosh & Goodman, 2016; Watson & Astor, 2025; Weist et al., 2018). However, much of this literature is conceptual or situates refugee students within broader culturally and linguistically diverse cohorts rather than as the primary focus of investigation. Consequently, evidence regarding the effectiveness, cultural responsiveness, and equity implications of MTSS and SWPBS for refugee populations remains underdeveloped.

Importantly, while these frameworks appropriately incorporate behaviour regulation to maintain safe and supportive learning environments, scholars caution that implementation lacking cultural responsiveness, relational understanding, and trauma-informed interpretation of behaviour may inadvertently marginalise refugee learners. This disparity between widespread framework adoption and limited refugee-specific evidence represents a critical gap in the literature, constraining understanding of how tiered academic, behavioural, and wellbeing supports can be adapted and implemented through trauma-informed, relational, and culturally sustaining approaches. Addressing this gap requires contextually grounded empirical research examining how multi-tiered frameworks can be tailored to support refugee students and strengthen inclusive and equitable schooling outcomes.

**Fig. 2:**
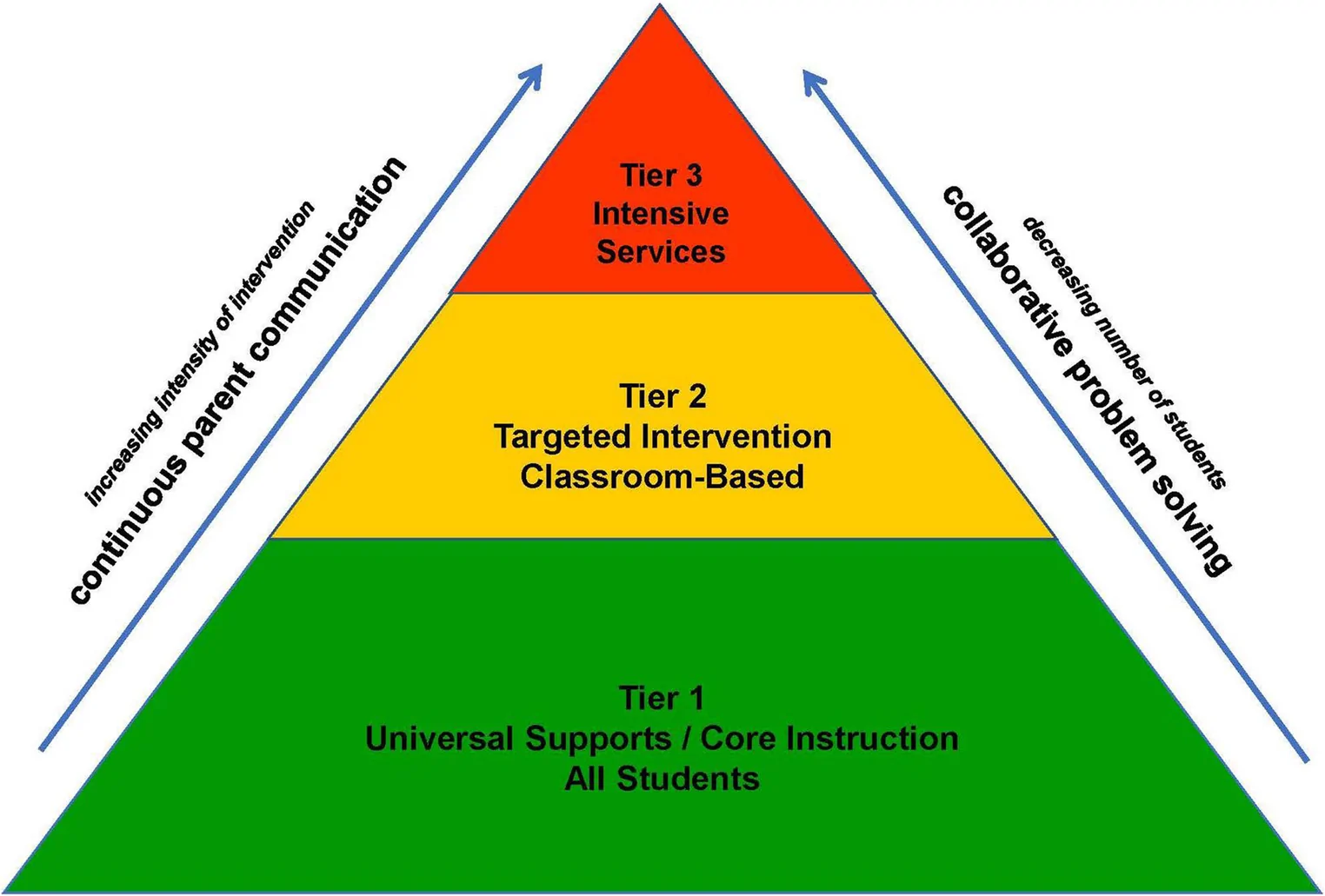
Multi-tiered system of support (MTSS) pyramid illustrating universal, targeted, and intensive interventions

**Figure ****2** illustrates a MTSS pyramid, showing how universal supports (Tier 1), targeted classroom interventions (Tier 2), and intensive services (Tier 3) are delivered with increasing intervention intensity, continuous parent communication, and collaborative problem-solving as the number of students requiring support decreases.

### Research Gaps

Despite growing scholarly attention, the literature on trauma-informed education for refugee students in Australia continues to reveal several persistent gaps across empirical evidence, systemic implementation, and professional learning. A central limitation identified in this review concerns the limited empirical examination of how MTSS and SWPBS frameworks have been applied within refugee education contexts. While trauma-informed principles are increasingly integrated into system level frameworks across other student populations, including Indigenous learners (Krishnamoorthy et al., 2024) and students affected by domestic violence (Davies & Berger, 2019), refugee focused scholarship has yet to systematically evaluate such intersections. Consequently, evidence remains underdeveloped regarding how multi-tiered systems can be adapted, implemented, and evaluated to support refugee-background students through trauma-informed, culturally responsive approaches.

Furthermore, most Australian studies remain descriptive and classroom-focused, emphasising relational pedagogy and culturally responsive practices while offering limited evaluation of institutional effectiveness or system-level implementation (Kaukko & Wilkinson, 2018; Miller et al., 2021; Wrench et al., 2018). The evidence base is also characterised by a scarcity of longitudinal and mixed-method designs, which restricts understanding of long-term outcomes and the causal impact of trauma-informed practices on student wellbeing and engagement (Barrett & Berger, 2021; Miller et al., 2018).

Significant gaps are also evident in professional learning and teacher preparation. Research indicates that educators often receive fragmented or one-off training with limited sustained institutional support, resulting in uneven application of trauma-informed principles across school contexts (Barrett & Berger, 2021; Odhiambo, 2025). These inconsistencies are further compounded by limited integration between trauma-informed pedagogy, culturally responsive practice, and schoolwide support frameworks, thereby constraining implementation fidelity and scalability (Lembke et al., 2024; Miller et al., 2021). Although MTSS and SWPBS are widely recognised as promising organisational structures for coordinating academic, behavioural, and wellbeing supports, empirical evidence examining professional learning for their trauma-informed implementation remains underdeveloped. International scholarship suggests that effective adoption of these frameworks requires sustained training, coaching, and leadership support to ensure culturally responsive and equity-oriented practice (Kim & Venet, 2025; McIntosh & Goodman, 2016; Weist et al., 2018). However, few studies explore how educators are prepared to integrate trauma-informed pedagogy within multi-tiered systems, particularly in refugee education contexts.

Compounding these challenges, policy misalignment between inclusion, wellbeing, and trauma-informed frameworks often produces fragmented practice and weak accountability structures (Miller et al., 2021). In addition, student voice remains notably marginalised, as much of the literature privileges institutional or teacher perspectives, leaving refugee students’ lived experiences underrepresented in research and policy discourse (Kaukko et al., 2021; Wrench et al., 2018).

Collectively, these gaps highlight the need for integrated, evidence-based, and participatory research that connects trauma-informed pedagogy with system-wide reform, sustained professional learning, and meaningful student agency. Addressing these limitations is essential to move beyond fragmented interventions toward a cohesive, culturally responsive, and equity-driven model of trauma-informed education in the Australian context.

## Discussion

This review demonstrates that trauma-informed pedagogy for refugee-background students in Australian schools is most effective when it is relationally grounded, culturally responsive, and systemically embedded. Across the reviewed studies, the convergence of relational, cultural, and structural dimensions of practice was evident, reflecting an emerging yet uneven field of educational reform. Consistent with (Barrett & Berger 2021; Kaukko et al., 2021), the findings reaffirm that empathy, trust, and emotional safety are central to enabling refugee students to recover, engage, and thrive in post-trauma learning contexts. The interpretations presented in this discussion are grounded in the ten studies included in this systematic review and should be understood within the scope and limitations of this evidence base.

There is, however, a growing shift from teacher-led innovation towards schoolwide and systemic approaches that embed trauma-informed principles within structured frameworks such as MTSS and SWPBS. Research suggests that these frameworks provide tiered mechanisms, including universal, targeted, and intensive supports, for integrating wellbeing, inclusion, and learning (Brown, 2025; Newton et al., 2023). Despite this potential, Australian research remains predominantly descriptive, emphasising relational pedagogy, classroom adaptation, and teacher perspectives while providing limited examination of system level implementation, program effectiveness, or longitudinal outcomes (Barrett & Berger, 2021; Kaukko & Wilkinson, 2020; Miller et al., 2018; Miller et al., 2021; Wrench et al., 2018).

Internationally, the field of trauma-informed education has matured towards policy coherence and system-level alignment, generating structured evidence of impact (Kim & Venet, 2025; Lembke et al. 2024; McIntosh & Goodman, 2016; Watson & Astor, 2025). In contrast, Australian research remains primarily practice-driven, characterised by relational commitment but limited policy evaluation, data collection, or longitudinal design (Barrett & Berger, 2021; Odhiambo, 2025). Consequently, trauma informed education in Australia is best described as a field of active innovation but partial consolidation, rich in empathy and classroom practice yet constrained by fragmented policy frameworks, limited evaluation, and inconsistent professional learning opportunities.

Building teacher capacity remains a key priority. Without sustained and contextually relevant professional learning, trauma-informed practice risks remaining inconsistent and dependent on individual initiative rather than institutional support (Barrett & Berger, 2021; Odhiambo, 2025). Furthermore, policy alignment between inclusion and trauma-informed frameworks is needed to ensure wellbeing and equity are pursued as interconnected rather than parallel initiatives (Liasidou, 2024; Miller et al., 2018).

Embedding trauma-informed principles within MTSS and SWPBS frameworks offers a promising pathway for strengthening system-level coherence. When implemented with fidelity and supported by culturally responsive and relational practices, these frameworks can enhance coordination across academic, behavioural, and wellbeing supports while promoting student agency, safety, and belonging (Kim & Venet, 2025; Thomas, 2025). Importantly, this interpretation reflects patterns within the reviewed literature rather than a generalised characterisation of schooling institutions.

While MTSS and SWPBS appropriately incorporate behaviour regulation to ensure safe learning environments, the reviewed scholarship cautions that implementation lacking cultural responsiveness and trauma-informed interpretation may risk marginalising refugee-background learners.

## Limitations and Strengths of the Review

This review is limited by the small number of eligible studies (*n* = 10), which restricts generalisability across Australian contexts. While predominantly qualitative designs provided rich contextual insight, they limited empirical precision and causal inference (Barrett & Berger, 2021; Kaukko & Wilkinson, 2020). Variations in how trauma-informed pedagogy was conceptualised across studies also reduced comparability. Some framed it as a relational, care-based stance (Baker & Naidoo, 2024; Barrett & Berger, 2021), others as a systemic inclusion framework (Miller et al., 2018, 2021; Odhiambo, 2025), and still others as a culturally grounded or identity-focused approach (Wiseman & O’Gorman, 2017; Wrench et al., 2018). This conceptual diversity, while enriching, hindered direct comparison of findings. Moreover, the lack of quantitative or longitudinal evaluations of MTSS and SWPBS frameworks constrains understanding of long-term impact and scalability.

Despite these limitations, the review offers several strengths. It provides one of the first systematic syntheses of trauma-informed pedagogy for refugee-background students in Australian schools, integrating psychological, pedagogical, and policy perspectives. Guided by PRISMA 2020 (Page et al., 2021) standards, the review maintains methodological transparency and employs inductive thematic analysis to capture relational, cultural, and systemic dimensions of practice. Its incorporation of multiple theoretical orientations, including trauma theory, ecological systems theory, social constructivism, and the ethics of care, enhances conceptual depth. Importantly, by identifying MTSS and SWPBS as emerging frameworks for systemic application, the review bridges classroom innovation with institutional reform and provides a valuable foundation for future policy and research.

## Future Directions

In light of the scope of the reviewed studies, recommendations are framed as directions for further research rather than prescriptive institutional mandates. Future research should prioritise longitudinal, mixed-method, and implementation-science approaches to evaluate the sustainability, fidelity, and scalability of trauma-informed multi-tiered interventions (Bauer et al., 2015; Newton et al., 2023). Policy-oriented research should also examine how trauma-informed principles can be embedded in educational leadership, funding, and curriculum design to ensure systemic sustainability (Liasidou, 2024; McIntosh & Goodman, 2016; Thomas, 2025).

Integrating neuroscientific, pedagogical, and relational perspectives will help bridge theory and practice, strengthening trauma-informed education as an evidence-based and contextually grounded framework (Kim & Venet, 2025; Perry, 2006). Moreover, student-centred and participatory methodologies are needed to amplify the voices of refugee learners, allowing them to co-shape practices that affect their educational experiences (Kaukko et al., 2021; Wrench et al., 2018).

Finally, linking trauma-informed pedagogy with systemic reform and multi-tiered implementation can move Australian education beyond isolated innovation toward a sustainable, equity driven model, one that empowers refugee and migrant background students to learn, belong, and thrive (Brown, 2025; Lembke et al., 2024).

## Conclusion

This review demonstrates that trauma-informed pedagogy for refugee-background students in Australian schools is most effective when relational care, cultural responsiveness, and systemic coherence are integrated. While progress is evident within the reviewed studies, implementation has primarily been examined at the classroom level, with limited empirical attention to system-level integration. Embedding these practices within structured, multi-tiered frameworks such as MTSS and SWPBS offers a scalable path toward equitable, whole-school reform. Sustained teacher development, policy alignment, and rigorous evaluation are essential for long-term impact. Future research should employ longitudinal and participatory methods to capture student perspectives and measure outcomes over time. Further empirical research examining trauma-informed education as a systemic, culturally grounded approach may strengthen understanding of how inclusion, belonging, and wellbeing can be enhanced for refugee and migrant-background learners across Australia.

## Data Availability

Data Availability Statement: Not applicable. No new data were created or analyzed in this study. Data sharing is not applicable to this article.
